# Leiomyosarcoma Ex Pleomorphic Adenoma of the Parotid Gland: A Case Report and Literature Review

**DOI:** 10.1155/2016/9795785

**Published:** 2016-09-08

**Authors:** Michael Coulter, Jingxuan Liu, Mark Marzouk

**Affiliations:** ^1^Health Science Center, Stony Brook University School of Medicine, Stony Brook, NY 11794, USA; ^2^Department of Pathology, Stony Brook University Hospital, 101 Nicolls Road, Stony Brook, NY 11794, USA; ^3^Department of Otolaryngology, Upstate University Hospital, 750 E. Adams, Syracuse, NY 13210, USA

## Abstract

There is only one previously reported incident in the English literature of sarcoma ex pleomorphic adenoma of the parotid and there are only 8 cases of primary parotid leiomyosarcoma. In our case, a 79-year-old female patient presented to our care with left preauricular pain, swelling, and facial weakness. After CT imaging, she underwent left total parotidectomy. A spindle cell lesion was identified intraoperatively and the facial nerve was sacrificed. Subsequent analysis of the lesion yielded a diagnosis of leiomyosarcoma ex pleomorphic adenoma. After 30 fractions of radiation therapy, scans were negative for tumor. However, 18 months after first experiencing symptoms, she was found to have metastases to the brainstem and lung. When diagnosing sarcoma ex pleomorphic adenoma of the parotid gland, it is important to perform thorough immunohistochemical staining and exclude a previous history of sarcoma or other sources of metastases. Complete resection is critical due to the tumor's local aggressiveness and metastatic potential. Although these tumors are not very responsive to chemotherapy or radiation, adjuvant treatment is commonly used when margins are unclear.

## 1. Introduction

Of all major salivary gland neoplasms, only 0.3 to 1.5% are diagnosed as sarcomas [1, 2]. Of the salivary gland sarcomas, malignant schwannoma and fibrosarcoma are the most common [2]. A rare occurrence is a primary leiomyosarcoma of the parotid gland, as only 8 cases [2–9] have been reported in the English literature, as detailed in Table 1. From this data, there seems to be no sex or age predilection; 4 were female and 4 were male with an average age of 40 ± standard deviation of 20.8 years. Leiomyosarcomas of the head and neck reportedly uncommonly metastasize to cervical lymph nodes and have a low potential for distant metastasis [4, 6]. However, 4 of the 8 primary parotid lesions reported metastases: 2 to lymph nodes, 1 to the scalp and lymph nodes, and 1 to the lungs [4, 6, 7, 9]. In order to diagnose a primary leiomyosarcoma of the parotid gland, Luna et al. proposed four basic criteria: (a) negative history of sarcoma at other sites must be excluded; (b) metastasis from the upper aerodigestive tract must be ruled out; (c) the macroscopic and histologic appearance must be consistent with an origin within the gland, instead of invasion by nearby soft tissue; (d) carcinosarcoma must be excluded by histologic analysis of multiple sections [1].

Leiomyosarcoma can have a primary site of origin anywhere in the body with smooth muscle. Although sparse in the head and neck region, smooth muscle is found mainly in the walls of blood vessels and erector pili muscles of the skin. Presenting symptoms of this tumor in the head and neck region are usually nonspecific and occurring due to mass effect. Histology and immunohistochemical studies are required for diagnosis. Leiomyosarcomas appear grossly hemorrhagic and soft. Microscopically, they exhibit pleomorphism and may show abnormal mitotic figures and coagulative tumor cell necrosis. Staining must be positive for smooth muscle markers such as smooth muscle actin and/or H-caldesmon, which excludes a myofibroblastic tumor [3]. They are nonreactive to S-100 and cytokeratin [4]. The mitotic activity could be helpful for determining malignancy as well as metastatic potential [10]. Based on the limited data, leiomyosarcoma of the head and neck behaves more aggressively locally and has a relatively poor prognosis [9, 11, 12]. Early surgical removal with wide margins has resulted in the best outcomes while the tumor is still small and in situ. These tumors are generally not very responsive to chemotherapy or radiation, although adjunctive treatment is still commonly used when margins are unclear or tumor cells are left behind.

Most of the salivary sarcomas and carcinosarcomas arise as de novo neoplasms. Pleomorphic adenomas may undergo malignant change to carcinoma ex pleomorphic adenoma, true malignant mixed tumor (carcinosarcoma), or metastasizing pleomorphic adenoma. Only one other case has been reported in which pure sarcomatous change was observed with no epithelial carcinomatous component and thus was diagnosed as a sarcoma ex pleomorphic adenoma [13]. In that case, a 59-year-old man presented with a parotid mass and subsequent FNA suggested findings of benign mixed tumor and thus a parotidectomy was performed. The tumor showed a high-grade sarcoma exhibiting metaplastic bone with evidence of vascular and capsular invasion within a background of an otherwise typical mixed tumor. Examination of additional sections demonstrated the same. The patient received chemotherapy after further workup demonstrated a recurrent and progressive disease with lung metastasis. The patient expired with complications 2 years after first presentation. The extremely rare occurrence of a leiomyosarcoma ex pleomorphic adenoma of the parotid presents not only significant academic interest and diagnostic conundrum but also important clinical support for aggressive management of this tumor with poor prognosis, comparable to all leiomyosarcomas of the head and neck.

## 2. Case Report

**Figure 1:**
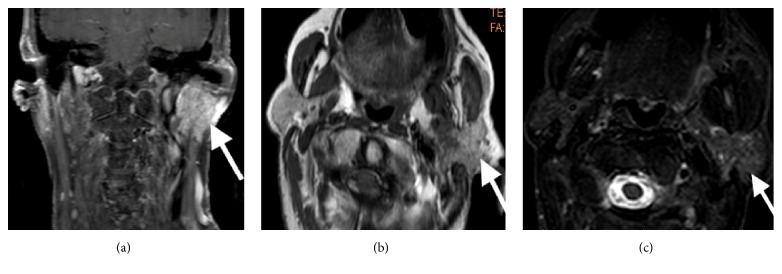
MRI revealing a mass in the deep lobe of the parotid gland abutting the posterior aspect of the lateral pterygoid muscle, likely extending towards the stylomastoid foramen exhibiting low T1 signal intensity in (a) coronal and (b) axial planes as well as low STIR signal intensity in (c) axial plane.

A 79-year-old female presented to the emergency department with 2 weeks of left jaw pain and swelling as well as left facial weakness and droop. As seen in Figure 1, an MRI with IV contrast revealed a 1.5 × 1.5 × 1.8 cm heterogenous, low-intensity, peripherally enhancing lesion located in the deep lobe of the left parotid gland, abutting the posterior aspect of the left lateral pterygoid muscle. It demonstrated likely pathologic involvement of the facial nerve within the parotid gland and in the region of the stylomastoid foramen. She was subsequently referred to our office and was found to have weakness of the marginal branch of the facial nerve and diminished gag reflex.

Due to high malignancy suspicion, a left total parotidectomy with facial nerve resection was performed as well as facial nerve reconstruction. The tumor was noted to be very firm and was encountered deep in the parotid gland, extending to the deep muscles in the neck but not adherent to any surrounding structures. The margins were distorted during the excision. Surgical pathology included 2 tumor excisions exhibiting spindle cell morphologies and 5 lymph nodes labeled benign. The first parotid excision was 2.5 cm and showed extensive calcification and hyalinization. The second was 1.9 cm, surrounded nerve bundles, and was noted to have focal necrosis. Diagnosis was a sarcoma ex pleomorphic adenoma with the sarcomatous component being consistent with a leiomyosarcoma.

The patient began external radiation 2 months postoperatively to the left total parotidectomy tumor bed using generous margins and tracing the path of the left facial nerve back to the stylomastoid foramen. Lymph nodes were not included since the surgical specimens were negative and spindle cell sarcomas do not generally metastasize to lymph nodes. Left facial droop improved over the course of 6 weeks of 30 radiation treatment fractions. Six months after surgery, PET scan of the head and neck showed no abnormal hypermetabolic foci within the head and neck region to suggest metastatic disease. Nonspecific bilateral hilar hypermetabolic densities were appreciated, so a repeat CAT scan 3 months later was recommended. At a one-year postoperative followup, the patient had no complaints and only marginal facial nerve palsy was noted on exam.

While vacationing in Florida, the patient presented to the local hospital with 2 days of symptoms including ataxia, diplopia in the right eye, and bilateral hand numbness. MRI revealed a homogenously enhancing mass in the left paramedian inferior pontine region of the brainstem measuring about 9 × 9 mm. Chest CT also revealed a lobulated 5.2 × 2 cm mass along the peripheral portions of the right upper lobe. The brainstem and lung masses were assumed to be metastatic sarcoma from the left parotid. She was not deemed a surgical candidate and was recommended to undergo palliative radiation therapy, but she preferred hospice care just 18 months after first experiencing symptoms.

### 2.1. Pathology Report

**Figure 2:**
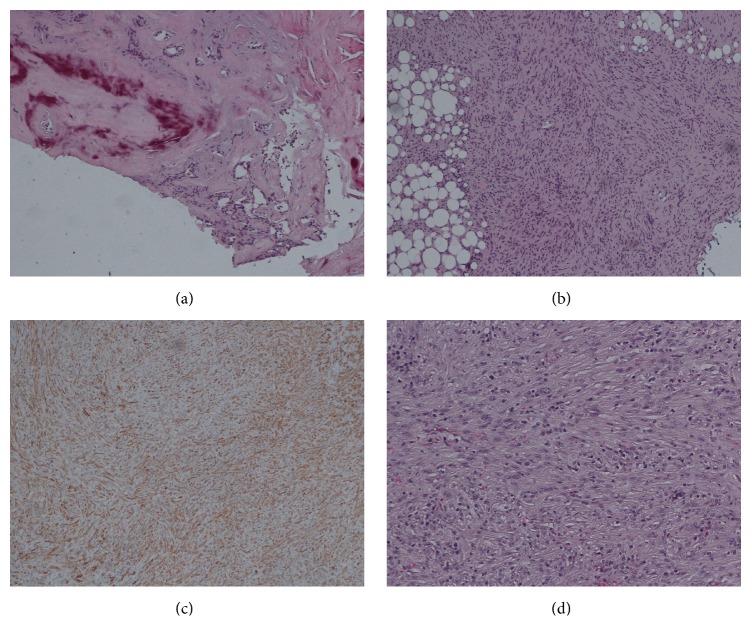
100x magnification of (a) calcifications around benign glandular tissue, (b) spindle cells infiltrating periparotid adipose tissue, and (c) smooth muscle stain of parotid lesion being positive. 200x magnification of (d) spindle cell lesion.

The parotid specimen was prepared on H&E and immunohistochemical stained slides, shown in Figure 2. Observations included the following: identifiable foci of pleomorphic adenoma with presence of benign glandular/tubular structures identified within a predominantly acellular hyalinized and focally calcified nodular area. There was also variably differentiated spindle-shaped cellular proliferation extensively involving periparotid soft tissues including perineural and perivascular invasion, as well as invasion of the parotid parenchyma. The lesion showed fascicular to storiform growth comprised of elongated cigar-shaped nuclei. There was increased mitotic activity including atypical mitoses and focal necrosis. The IHC stains were variably reactive for smooth muscle actin, smooth muscle myosin heavy chain, desmin, and calponin but negative for pan-cytokeratin, S100 protein, CD34, and ALK1. This is consistent with leiomyosarcoma. While there was focal moderate nuclear pleomorphism, the nuclear morphology was relatively bland lacking features of a histologic high-grade neoplasm. Diagnosis is a sarcoma ex pleomorphic adenoma with the sarcomatous component being consistent with a leiomyosarcoma. The absence of epithelial malignancy precludes a diagnosis of carcinosarcoma. The specimen was defined as histologic grade 2, including perineural and perivascular invasion with tumor extension into periparotid soft tissue. 12 lymph nodes were examined and 0 were noted for malignancy. Thus, the stage of the tumor was T_1_N_0_M_0_.

## 3. Discussion

Our case would be only the second reported sarcoma ex pleomorphic adenoma and is unique because of the sarcomatous component being consistent with leiomyosarcoma. A primary leiomyosarcoma of the parotid gland is also extremely rare. Thus, even after thorough H&E and immunohistochemical staining, this case is very difficult to diagnose. The differential is vast and the possibility of metastasis must be ruled out. Other possibilities include carcinosarcoma, sarcomatoid carcinoma, melanoma, and sarcoma of any origin. Also, it is important to suspect a metastasis from a primary site more commonly home to these neoplasms, such as the uterus in females. Saiz et al. presented the case of a uterine leiomyosarcoma that metastasized to the parotid gland 6 years before any clinical presentation in the uterus [14].

Although smooth muscle cells have been thought to be the origin of leiomyosarcomas, some authors have claimed that they may derive from pluripotent mesenchymal cells. When leiomyosarcomas of any anatomic site metastasize, they usually do so hematogenously as observed in 20% of cases. Lung involvement is seen in about 75% of cases due to the pulmonary vascular bed's filtering function [6]. As observed in the limited number of cases of primary parotid lesions, they seem to disseminate hematogenously and lymphatically. Local recurrence was only seen in one case, where it recurred thrice.

## 4. Conclusion

Leiomyosarcoma ex pleomorphic adenoma and primary leiomyosarcoma of the parotid gland are extremely rare occurrences, and diagnosis is challenging. It is important to perform thorough immunohistochemical staining and exclude a previous history of sarcoma or other sources of metastases, such as the upper aerodigestive tract or uterus. As with other sarcomas of the head and neck, complete resection of the primary tumor with tumor-free margins is critical especially due to the local aggressiveness and metastatic potential.

## Figures and Tables

**Table 1:** Literature review of eight primary parotid leiomyosarcomas.

Study	Age (years)	Gender	Presenting symptoms	Metastases	Treatment	Local recurrence	Followup
[2]	59	Female	Not reported	None	SX, RT	No	In remission 9 years postop.
[3]	78	Male	Painless mass	None	SX	No	In remission 5 years postop.
[4]	8	Male	Painless mass	Lungs	CRT	N/A	Lost to followup
[5]	45	Female	Painless mass	None reported (only local extension)	RT	N/A	Expired 5 years after presentation. Tumor directly extended into ipsilateral temporal lobe penetrating the orbit, maxillary sinus, zygomatic arch, ethmoid bone, sella turcica, and nares
[6]	33	Female	Painless mass	Lymph nodes & scalp	SX, RT	No	In remission 5 years postop.
[7]	17	Female	Painful mass	Lymph nodes	SX (three times, the last included excision of SCM and block dissection of cervical lymph nodes)	Thrice	Not reported
[8]	44	Male	Painless mass	None	SX, RT	No	In remission 3 years postop.
[9]	36	Male	Painful mass	Lymph nodes & facial nerve	SX, CRT	No	Not reported

SX: surgical excision; RT: radiation therapy; CRT: chemoradiation; N/A: not applicable.
